# Valedictory Address, Atlanta Medical College

**Published:** 1874-04

**Authors:** Wm. R. Burgess

**Affiliations:** Macon, Ga.


ATLANTA

MEDICAL AND SURGICAL JOURNAL.

VOL. XII.] APRIL—1874. [No. 1.

### Original Communications.

### VALEDICTORY ADDRESS.

DELIVERED BEFORE THE GRADUATING CLASS OF THE ATLANTA MEDICAL COLLEGE, WEDNESDAY EVENING, MARCH 4, 1874.

BY WM. R. BURGESS, M.D., MACON, GA.

*Gentlemen:* Having been honored with the high compliment of an invitation from your distinguished faculty, to deliver the valedictory address to the graduating class this evening, allow me to assure you that it is with feelings of unaffected distrust in my humble capacity to do justice either to you or to this interesting occasion, that I proceed to discharge the pleasing duty.

For so long a time, you have been listening to the voice of wise counselors and faithful teachers, now that you have reached the goal for which from your earliest pupilage you have so faithfully labored and looked forward to, with feelings mingled with fear and hope; standing at this time, as you do, the peers of your late teachers; the time having come when you are soon to say the sad farewell to instructors, friends, and beloved foster mother; it is but reasonable that you should at this closing moment expect to be entertained by a discourse teeming with beautiful rhetoric and sound philosophy. However far, gentlemen, I may fall short of this natural expectation, I shall rest in the abiding confidence that your charity will cover all.

The profession of medicine—that which you have chosen for your future labors, both mental and physical—reaching, as it does, far back into remote ages of antiquity; emanating from its shadowy, chaotic origin at a time when priestcraft reigned supreme; advancing and declining by turn, century after century, through multiplied and multiform vicissitudes that befell it on every hand, yet gaining new strength and new advocates until the earliest dawn of literature, art and science; then coming forth from the cloister and monastery, with substantial merit equaling any of the kindred sciences, is a theme well worthy your most ardent zeal and profoundest regard.

You have to-day, for the first time, gentlemen, had your names enrolled, and will in future, from your own choice, take your place in the arena of this long-existing and still raging contest. How much of valor and of scientific strategy, how much you are as individuals to accomplish towards driving the enemy from the remaining strongholds now under his dominion, is very much for your own choosing. Thus far you have been in the camp of drill, or instruction, only "snuffing battle from the distance." To-day your faithful and efficient officers have brought you forth well drilled and equipped, to see how valiantly you are to contest for victory on the field of actual conflict.

When first clothed with the doctorate, it is doubtless sometimes the case that the young physician feels no longer a necessity for study; feels that the few years already spent in laborious and close application to theory, as laid down in the books, or taught in the schools, entirely qualifies him to go forth and do battle against the invasion of disease, without the necessity for further reading; or, perhaps he feels strong in the confidence reposed in the power of his native genius to elucidate, and make plain to his understanding, all the obscure, and may be, enigmatical problems, that are destined at times to claim his attention in the sick-chamber. If such a feeling is cherished, if such confident expectation is entertained, by any of those whose bright, intelligent faces it is my privilege to look upon this evening, let me warn you at the outset of this deadly "Upas." The profession you have chosen—the science which is now advancing to its grand consummation, is constantly undergoing modification and change in its onward march, and will require, nay demand of its followers, unabated zeal in tracing out the published opinions and discussions of its eminent advocates, in every continent, land and country on the face of the globe, if he has at heart the good of his patient, or a desire to stand above mediocrity in the ranks of his co-laborers. A distinguished friend, in delivering an address before a graduating class of this college fourteen years ago, gave this excellent advice. He says: "aiming high, gentlemen, the achievement of science will require that your efforts shall be worthy of, and commensurate with, your aspirations. You must, through the powers and energies of mind and body, be the architects of whatever of fame and fortune you shall realize. Success will not result from accident, chance, or the favoring influence of external circumstances, but must be looked for in the manner in which you shape your conduct and character—in studious habits, industrious labors, energetic action, observation of the useful, self-control, honorable conduct, and generous benefactions, the highest reward of which is the rich and glowing consciousness of having done good. Your intellectual attainments and moral qualities will mark out your prominent professional position, uninfluenced to any considerable degree by the adventitious aids of fortune, family, friends, or social rank." Fixing then, the important fact in your minds that you hold your destiny in your own hands, that it will not do to trust the honor of your name and reputation to the influence of wealth, family or friends, but conscious of the power that lies within the grasp of your own intellect, "suffer not the blighting mildew of indolence, the syren voice of pleasure, an interest in other business avocations, the labors and cares of even a large practice, nor the witching smiles or allurements of the hour, to turn you from your purpose—your love of study. But procuring, in the outset of life, a small and well-selected library of standard authors, replenish it from time to time with valuable monographs as they fall from the press, and subscribe for a few good periodical journals; and, for years to come, ever arranging your hours of business and pleasure with an eye to the improvement of your mind, and the acquisition of knowledge, master and treasure up from the best authors, the rich and varied stores of existing information; evoke by your own thought from the great laboratory of nature, some of her yet hidden mysteries; and peruse the monthly and quarterly periodicals with sufficient regularity to enable you to keep pace with the progress of the science. In your reading let not the false glare of novelty with its dazzling fallacies captivate, neither let pre-conceived views or blind obedience to the *dicta magistra* obscure your mental vision to the importance of new light; but being cautious in adopting that which purports to be new, and still more cautious in rejecting that which has borne the test of long experience, and yet commands the confidence of the well-informed; contrast the views of one author with those of another, and then again with what you may have yourselves observed at the bedside. Thus you will pursue the path of safety, intelligence and certainty, and accumulate a fund of solid and valuable information co-extensive and co-temporaneous with the scope and uttermost progress of your art, that will be a tower of strength—the Archimedean lever with which you will be able to prize up the valuable truths of science; the key with which you can unlock the great arcana of disease. It will be the means of giving you promptness, decision, confidence and energy, as practitioners, and commanding for you success in treating the numberless maladies in their many varying and protean forms, to which both mind and body are subject."

But, gentlemen, valuable as intellectual culture may be—yea, essential and absolutely necessary to high position in the profession—this alone will by no means secure its attainment. The effulgence of genius and learning may for a time, as the lightning's flash or meteor's glare, captivate the beholders; but this, unsupported by other virtues, will, like the meteor's flash, charm but for a moment. It cannot command for any considerable length of time, unreserved confidence, lasting success, or distinguished eminence, nor commend you to the good, the virtuous and the pure.

"To secure this there must be associated and combined with general and extensive mental acquirements, the virtues of a moral character unstained by the vices, the immoralities and dissipations that so frequently attach to and wreck the promise of genius. The physician who would establish himself firmly in the confidence and affections of the public, and plant himself on an elevated platform in a profession illustrious in all ages for its distinguished exemplars of all the cardinal virtues of the heart, even as for their great learning, must sustain a moral character free from spot or blemish. Invited and admitted into the sacred precincts of domestic life, behind the scenes that shut off the world from the weaknesses and frailties of mortality divested of its mask of diplomacy and disguise; the trusted adviser and chief hope of man in his affliction and anguish, having committed unreservedly to his keeping communications involving interests and honor the most inviolable; stories of suffering and woe, mental and physical of all ages, sexes, and ranks; enjoying as he does a confidence and toleration entirely unknown to any other member of a community; the safety of society, the sacred observance of the trust reposed, and the very instincts of nature, demand that he should be a man entirely true and faithful."

Using the language of an eminent teacher: "As ministers of grace to your fellow-men, you go out from these halls to-day as apostles in a true sense; men sent out, commissioned for especial service, by highest human laws, even the authority of a sovereign State. You are sent out to fill the gaps left by faithful servants, who having "finished their course" do now "rest from their labors." "You go out as guards for the masses—guards for society and guards for your country. Not as scientists of war to destroy, even though they may destroy to save; your mission is to save and not destroy." Who can conceive of a higher, nobler calling than this? A community of young men, the flower and pride, yea, the very hope and promise of the country, giving themselves to years of earnest, determined, continuous study, burning the midnight lamp, even to the "we sma' hours" of morning; then with arduous labor bending over the cadaver, intently investigating that they may be correctly informed of their own mysterious, yet beautiful and complicated mechanism; anon wending their way with preceptor or clinical instructor through the cheerless wards of the hospital, taking note here of disease in some one of its multiform phases, then of some other malady; and again, assisting the surgeon in applying the ever ready knife for the removal of some noxious foreign growth, or mangled limb; and all, all, for what! Is it for gold? Not by any means. Ah! these years of study and of toil both of mind and body have been passed, not in the accumulation of gold, but in the spending of treasure with prodigal hand. Possibly there may be some in the graduating class of this commencement, who have cherished the illusion that this investment will return to them in due season in the form of handsome dividends. If there be any such in my auditory, if there be a member of this class whose aims and objects aspire to nothing beyond the amassing of fortune, I would make haste to warn him of the ignis fatuus he is pursuing, and advise a speedy change of tactics, in order that he may seek a more direct and certain route to the goal of his ambition.

"The lust of gold succeeds the lust of conquest;
The lust of gold unfeeling and remorseless!
The last corruption of degenerate man."

It is for such that the field of quackery, of charlatanry, of the numerous "isms" and "pathies," have special charms. Man is philosophically a strange and mysterious mingling of the "spiritual and temporal"—of the "material" and "immaterial." Hence we may not be surprised in occasionally finding the sensualities running out in search of their kindred spirits. For such, I cannot do better than repeat to you what a distinguished professor said to his class: "Deal with them personally with that courtesy of which a gentleman should never fail; but professionally, touch them not. Give professional place to them? No, not for a moment; counsel not with them under any circumstances: there can be no profitable conference, for there can be no compromise between truth and error."

True, it is sometimes an embarrassing matter for the young practitioner to deal thus with such professional "monstrosities." Self-interest, personal comfort, a misconception of what may infringe on the feelings of some person or persons, other than the direct violator of professional ethics, strongly incline one to overlook these irregularities. But when

"Grizzling hair the brain doth clear,"

no manner of preferment, present or prospective, will stand as a tempter in your way. No fear of professional or social injury, no loss of popularity, nor any other influence will tend to swerve you from the pathway of duty and of honor.

"Stern daughter of the voice of God!
O duty! if that name thou love,
Who art a light to guide, a rod
To check the erring, and reprove;
Thou who art victory and law,
When empty terrors overawe,
Give unto me, made lowly wise,
The spirit of self-sacrifice."

Fortunately for the good of science, the class of practitioners just alluded to, those whose highest, if I may not say only aspiration, is self-aggrandizement, constitute the few, whilst the many cherish the ennobling virtue of the largest and most self-sacrificing philanthropy. For all such, gentlemen, the profession of medicine is full of charms and freighted with attractions; for all such the field for emancipating humanity from the thraldom of anguish, misery and woe, both physical and mental, is ever open, enlarging and expanding with the march of conquest and civilization, inviting the laborer to his work. In this field he finds material for the fullest and most complete exercise of every faculty of his elastic and outreaching intellect. Hence it is that he embraces the opportunity of bringing into practical exercise, the well-learned and carefully-considered lessons of his pupilage; here, too, he has the privilege of verifying or refuting theory by practice, of testing the opinions of others by absolute personal observation. Here it is that his observing, philosophical mind may discover and bring to light new and important truths hitherto undiscovered. To such an one, the observance of the struggle between disease and health is full of deepest interest; the one tending to pervert and derange, whilst the other is ever struggling to drive out the invader, and keep in perfect play all the functions of the organism; the one tending to destroy life, the other battling for its preservation. Here it is that he may watch, observe and note the power and efficacy of his remedies.

In this busy, practical school he will find an opportunity, if not a demand, for bringing into exercise all the cardinal virtues of his character. Self-control, an absolute necessity to successful ministration, will serve him as a well tried friend. Very much will be constantly occurring in this practical life, in this experience of enslavement to duty, duty both to your own fair name and reputation, and to the sacred trust committed to your keeping, that will necessitate the calling to your aid, patience in her most "perfect work." Few there be whose trials are more numerous than those of the physician, but the school of experience will soon learn you, that

"In your patience you are strong."

Disappointment, too, will often be your guerdon. Death has ever claimed its victims, and so will continue to do to the end of time. It has been the lot of the most devotedly zealous, learned and skillful physicians occasionally to see the ravages of disease prove intractable to their remedies; and, gentlemen, it will be yours. Sad as it is to sit by the dying bed of those appealing to you, as their preferred hope of safety, in time of distress and danger; sad as it is to behold the look of despair and anguish in the imploring faces of the loved ones of your fast-sinking patient, as they all, with common voice, appeal to you for help—I say, sad as all these things may be, you should bear in mind that they most assuredly await you. When (as will sometimes be the case) you are summoned to the sick room, and your well-trained faculties behold at a glance, from the beginning, the sad end of your patient, fain would you screen yourself behind some pretext to throw the responsibility on older shoulders, but for the consequences that might result to your reputation by such a course. Young gentlemen, don't yield to this alluring temptation; do not desert your post in time of peril: it is cowardly and censurable in the extreme. Do your duty, your whole duty, and leave results to a higher power. In the faithful discharge of duty, even under these adverse and gloomy circumstances, perhaps as much as when skies are bright and more propitious breezes glide your little bark smoothly and silently along without a billow or a breaker to disturb, you are weaving for yourselves imperishable garlands, that will remain with you, green and fragrant, to the close of your lives.

Your qualifications for going out into this new life which you have to-day assumed, to practice your responsible profession, the high character and distinguished eminence of your late teachers abundantly attest. Do not suffer them to feel disappointment's sting, because of your failure to come up to, and fill the measure of, their brightest hopes. Select some one from the ranks of the profession, either of the dead or the living, some individual whose character, in all of its aspects, you would be proud to emulate, and make him the beau-ideal of your aspirations, remembering that

> "In the lexicon of youth,
> Reserved by fate for a bright manhood,
> There is no such word as fail."

There is not the shadow of a reason why you should not attain in your profession to any degree of eminence that a reasonable ambition may aspire to. If you have not already done so, read the discourse delivered by Prof. Gross before the class of Jefferson Medical College last year, on the "Life and Character of Ambrose Pare," and you will at once see that the early life of this illustrious Frenchman afforded him most unpromising resources. Yet he rested not until by his energy and exertions his name was recorded among the great leaders of his art. And so it has been in innumerable instances in every department of life. The hope of the community, and the highest expectation of your friends, are with you. This large auditory of intelligence, worth and beauty, fully attests the deep interest that is felt in your behalf. Then, gentlemen, with the benedictions of the good and pious, the urgent appeals from your friends, and beyond and above all, the smiles of lovely woman resting upon you, and, like the noble Spartan mother, bidding you go forth as invincible conquerors, how can you do else than, like the Spartan youth, resolve that you will return in triumph, or perish at your post!

In closing, I would urge you to be ever diligent in your good work. While you contemplate what may be before you in life, carefully review the past, think of the long ages of patient toil it has required to bring medicine to its present high position—ages during which multitudinous elements of superstition, wrath and venom stood as mountain barriers to oppose. Trace it along its fluctuating journey to the beginning of that long and dreary night that brooded over the civilized world. Then, after this morbid slumber of a thousand years—years when naught possessed the souls of men but terror and alarm at the carnage of bloody, desolating wars that swept like a besom of destruction over the learned world: see it at the earliest revival of literature, marshaling the remnant of power and life that remained, coming forward to take position in the front rank of sister sciences. Remembering the long-continued and hard and nobly-fought struggle it has already cost; and remembering, too, that we are still on the advance march, not yet having vanquished the last foe, buckle on your armor with right good will and, emulating the illustrious host who now rest from their labors, with commendable rivalry for the immense retinue of genius and learning who are to-day pioneering in every section of the world, be not content till each of you shall have contributed his prorata share towards the glorious consummation of the perfection of medical science.

---

The Archives of American Medicine and Surgery, volume No. 1, published by the Faculty of the College of American Medicine and Surgery, at Macon, Georgia, is about the most crude attempt at a medical journal that has come to hand. Many pages are upside down, and the matter of the journal, like the faculty of the college, seems to have been gathered in from all kinds of sources.—*The Clinic.*

